# Pan-serological antibodies and liver cancer risk: a nested case-control analysis

**DOI:** 10.1038/s41598-025-89629-2

**Published:** 2025-02-14

**Authors:** Cody Z. Watling, Xing Hua, Jessica L. Petrick, Xuehong Zhang, Whitney L. Do, Limin Wang, Evan Maestri, Kai Yu, Xin Wei Wang, Katherine A. McGlynn

**Affiliations:** 1 Division of Cancer Epidemiology and Genetics, National Cancer Institute, Bethesda, MD USA; 2 Slone Epidemiology Center at Boston University, Boston, MA USA; 3 Department of Medicine, Brigham and Women’s Hospital, Harvard Medical School, Boston, MA USA; 4 Department of Epidemiology, Harvard T.H. Chan School of Public Health, Harvard University, Boston, MA USA; 5 Laboratory of Human Carcinogenesis, Center for Cancer Research, National Cancer Institute, Bethesda, MD 20892 USA; 6 Liver Cancer Program, Center for Cancer Research, National Cancer Institute, Bethesda, MD 20892 USA; 7 National Cancer Institute, 9609 Medical Center Drive, Rockville, MD 20850 USA

**Keywords:** Antibodies, Hepatocellular carcinoma, Prospective, Cohort, Viruses, Bacteria, Cancer epidemiology, Immunology

## Abstract

Recently, studies have reported that pan-viral serology signatures may be predictive for liver cancer development. However, whether these same findings are observed for prospective studies has not been previously investigated. The nested case-control analysis included 191 persons who developed liver cancer and 382 controls from the PLCO prospective cohort. The presence of circulating antibodies, measured by VirScan, was determined in serum samples obtained at study recruitment. The presence of antibodies was compared between cases and controls using multivariable conditional logistic regressions, and prediction models were used to estimate whether exposures predicted liver cancer development. No significant associations were found between antibodies to viruses, bacteria or allergens and liver cancer risk after adjustment for multiple testing. The agent most significantly associated with risk was hepatitis C virus (HCV), but it was only detected among 23 participants (odds ratio (OR): 3.98; 95% confidence intervals (CI):1.59–9.99; *p* = 0.0032, False Discovery Rate (FDR) = 0.35). In prediction models based on 109 antibody features, no associations with liver cancer risk were observed (area under the curve [AUC]: 0.52–0.54). In analyses restricted to the most common type of liver cancer, hepatocellular carcinoma, the association with HCV was stronger (OR: 23.16, 95% CI: 4.55-117.68; FDR p-value = 0.0016), although prediction models based on all detected antibodies were similar (AUC = 0.55; 95% CI:0.43–0.68). Antibodies to no infectious agents, other than HCV, were found to be prospectively associated with liver cancer risk. The utility of using an antibody exposure signature prospectively for liver cancer development needs to be further explored.

**Supplementary Information:**

The online version contains supplementary material available at 10.1038/s41598-025-89629-2.

## Introduction

Liver cancer is the third most common cause of cancer death worldwide^1^. In the United States, the 5-year relative survival is only 20%, resulting in liver cancer having the second highest mortality rate after pancreatic cancer^2^. The dominant histological subtype of liver cancer in almost all countries, including the United States, is hepatocellular carcinoma (HCC). Chronic infection of hepatitis C virus (HCV) or hepatitis B virus (HBV) are well established causal risk factors for HCC^3,4^. Other major risk factors include excessive alcohol intake, smoking, consumption of aflatoxin B_1_ contaminated foods, and metabolic conditions including metabolic syndrome, obesity, diabetes, and metabolic dysfunction-associated steatotic liver disease (MASLD)^3^. These factors, and some rare genetic disorders, collectively account for ~ 60% of HCC in the United States^5^ suggesting the existence of other possible factors.

A possible factor that may influence liver cancer risk is an individual’s history of exposure to infectious agents. It is estimated that 13% of global cancer incidence is attributable to infectious pathogens and viruses^6^. These agents individually, or collectively, may influence carcinogenesis by altering host immunity or disrupting normal cellular processes^7^. HCV and HBV both cause inflammation that can lead to chronic liver disease^4,8^; however, recent studies have suggested that other viral exposures may also be associated with liver cancer^9–11^. A case-control study conducted in the United States reported that a particular viral exposure signature, containing unique epitopes of 61 viral strains (some of which were HCV and HBV), was more common among HCC cases than controls^10^. Another recent case-control study conducted in Thailand, which included 663 cases of HCC and 686 population controls, found that 46 viral features (of which 13 were HCV or HBV-related) were more common among HCC cases than controls^11^. As blood samples in both studies were collected from individuals at the time of HCC diagnosis, it was not possible to determine whether the viral signature was related to the development of HCC or was a consequence of having HCC. As such, whether a history of exposure to infectious agents is prospectively associated with risk of developing liver cancer remains unclear.

In the past, examining infectious agent exposure history was challenging as assays had to be run on an agent-by-agent basis. Recent advancements in technology, however, have made it possible to assess lifetime exposures in blood samples using synthetic virome technology. VirScan^12^ utilizes phage immunoprecipitation sequencing technology, which displays proteome-wide peptides, to detect antibodies against viruses, bacteria, parasites, and common allergens. Antibodies to 206 species that are known to infect humans, and over 1000 strains, can be detected using VirScan^12^. The objective of this study was to examine whether a history of infectious agent exposure, measured by VirScan, was associated with risk of developing liver cancer in a nested case-control study in the Prostate, Lung, Colorectal, and Ovarian (PLCO) Cancer Screening Trial. To accomplish this, we explored if there were differences and associations in detected antibodies between liver cancer cases and controls. We also attempted to replicate results from previous case-control studies^10,11^, which reported that serological exposure signatures can predict liver cancer, in a prospective cohort study.

## Materials and methods

### Project design

The PLCO study has been described in detail elsewhere^13,14^. Briefly, PLCO was a multicentre trial assessing the effect of screening for prostate, lung, colorectal and ovarian cancers on cancer mortality. Individuals between the ages of 55 and 74 years were recruited between 1993 and 2001 from 10 screening centers in the United States. Approximately 155,000 participants enrolled in the study and were randomized to either the screening arm (*n* = 77,000) or to the non-intervention arm (*n* = 78,000). At recruitment, participants provided informed consent and completed questionnaires regarding their health history, demographics, and lifestyle factors. In addition, non-fasted blood samples were collected from the participants in the screening arm. The samples were stored at -70 °C.

As the current project necessitated the use of blood samples, the participants were selected solely from the screening arm of the trial. All 191 persons who developed liver cancer were matched to two controls each, resulting in 382 controls. All controls were alive and free from cancer at the time of their matched case’s diagnosis. The matching variables were age (+/- 1 year), race/ethnicity, sex, and date of baseline blood collection (+/- 3 months).

Diagnoses of liver cancer were ascertained via linkages to state cancer registries. Liver cancer was identified using the International Classification of Disease (ICD) 10th edition code: C22. If available, ICD-O-3 morphology codes were used to determine liver cancer histological subtypes, specifically HCC: 8170–8175 and intrahepatic cholangiocarcinoma (ICC): 8032–8033, 8041, 8050, 8070–8071, 8140–8141, 8160, 8260, 8480, 8481, 8490. The median time from blood collection to diagnosis of liver cancer was 10.8 years (IQR: 6.3–15.1).

## Study and project approvals

PLCO originally received ethical approval from the NCI Division of Cancer Prevention and Control advisory Board of Scientific Counsellors in January 1991. The study was conducted in accordance with relevant guidelines and regulations. The present project protocol and use of data was approved by the PLCO steering committee (project number: EEMS-2021-0015).

## Laboratory analyses – VirScan

Infectious agent exposure history was determined using VirScan^12^. VirScan is a technology, used to analyze antibodies against viruses, bacteria, parasites, and food allergens in human samples using phage immunoprecipitation and has been described in greater detail elsewhere^12^. Briefly, the T7 bacteriophage used by VirScan displays peptides from 206 species and over 1000 different strains of infectious agents providing proteome-wide coverage of all viruses known to infect humans as well as many bacterial proteins and food allergen antigens. Specific laboratory methods related to T7 phage library proliferation and library quality control were employed as previously described by Liu et al.^10^. VirScan phage immunoprecipitation sequencing also followed the methods described in detail by Liu et al.^10^. Briefly, serum samples were mixed with the bacteriophage library and rotated overnight. The following day, magnetic beads were added to each well and allowed for immunoprecipitation. After 4 h, the beads were washed three times with wash buffer and the beads were then resuspended in water and lysed. The lysed phage material then went through two round of polymerase chain reaction amplification and were indexed and combined for sequencing using the Illumina NextSeq 500. All samples were run with technical duplicates and each plate included six empty wells (negative controls) and three wells filled with a single control sample from one individual which is utilized across plates. A total of 626 serum samples were analysed consisting of 191 cases, 382 controls and 53 quality control samples.

## HCV and HBV serology

Serum samples from both cases and controls were tested for HBV and HCV. To determine HBV status, hepatitis B surface antigen (HBsAg) was assayed using HBsAg 3.0 enzyme immunoassay (Bio-Rad Laboratories, Redmond, WA, USA). To determine HCV status, antibody to HCV was assessed using Ortho HCV Version 3.0 enzyme-linked immunosorbent assay (Ortho-Clinical Diagnostics, Inc.).

## Statistical analyses

Normalization of data derived from VirScan has been previously described in detail^10,15^. Briefly, for every epitope in VirScan, we calculated enrichment Z-scores to determine if an epitope was present (i.e., presence of an antibody to a particular epitope) and to quantify relative magnitude of enrichment of each epitope. Peptides were binned in descending rank order based on the estimates from the negative control (VirScan estimations performed without adding any blood sample) with a bin size of 300. For each sample, read counts for each epitope were grouped into their predefined bins and the top and bottom 5% of epitopes were temporarily removed to determine the mean and standard deviation for Z-score calculations. Presence of an antibody against a specific epitope was defined as immunoprecipitation of an epitope with a Z-score > 6.5 on both plates for an individual peptide. Antibodies can cross-react with similar peptides from related viruses. To address potential cross-reactivity among VirScan peptides, in any instance where a species had a shared subsequence of seven amino acids present in individual samples, only the epitope from the antigen that had the greatest number of enriched peptides across different species was counted. The epitope binding signal scores were averaged and log-transformed across replicates. An organism was classified as seropositive if any number of peptides were enriched.

Baseline demographics of participants were summarised by case and control status. Prevalence of viral species/organisms, bacterial species, or other antibodies detected were compared between cases and controls using chi-square tests. We also compared the presence of antibodies across covariates (e.g., sex, race/ethnicity, body mass index) using chi-square tests, ANOVAs, or t-tests. We compared the number of antibodies (primarily viral species) detected in HCV(+) and HCV(-) participants. Multivariable conditional logistic regression was utilised to estimate odds ratios (OR) and 95% confidence intervals (CI) between viral infection species and organisms, bacterial species, or allergen antibodies and liver cancer risk conditioning on age, sex, ethnicity, and time of blood draw, and additionally adjusting for body mass index (BMI), education, smoking status, diabetes status, alcohol intake, and coffee intake. There was relatively low amount of missing covariate data (< 1%) for most of the covariates, except for alcohol intake and coffee intake in which 8% had missing information. Participants with missing covariate data were randomly assigned to categories for categorical variables (education, smoking, diabetes) or the mean was used to replace the missing value for continuous variables (BMI, alcohol intake, and coffee intake). No participants had any missing data on detected antibodies. We used Benjamini-Hochberg procedure to control the false discovery rate (FDR) in multiple testing.

Focusing on the antibodies with a prevalence of greater than 1% in the study samples (*n* = 109 antibodies) we conducted unsupervised hierarchical clustering to explore the relationships between samples based on their antibody profiles. To do this, each participant was assigned a vector of 109 binary variables with each variable representing one of the top antibodies. Participants were assigned a value of 1 for an antibody if its corresponding Z-score exceeded a predefined threshold (> 6.5), and 0 otherwise. We defined the distance between pairs of samples using the L1 norm of their binary vectors. Hierarchical clustering was performed using the Ward method, implemented through the R package ‘hclust’. We also explored differences between cases and controls using principal component analysis of the top 109 antibodies, using the R package ‘prcomp’.

To attempt to replicate previous findings^10,11^, we employed three different strategies to build models using the top 109 detected antibodies to discern between case and control status. These strategies include LASSO, Random Forest, and XGBoost, which are common choices for handling a relatively large number of predictors^16–18^. We determined the tuning parameters for each model using Monte Carlo cross-validation with 1000 simulations. In each simulation, 500 samples were randomly allocated to the training set, and the remaining 73 samples were assigned to the testing set. To adjust for confounders, samples were weighted using inverse probability weights based on participants confounder data. The performance of the models in the testing set was compared using the Area Under the Curve (AUC) and 95% confidence across the 1000 simulations.

### Sensitivity analyses

In sensitivity analyses, the outcome of interest was restricted to HCC. We reran analyses looking at prediction models for only HCC cases and compared presence of antibodies between the cases (*n* = 119) and non-cases (*n* = 238). We did not assess ICC cases in separate analyses due to the small number of cases (*n* = 38). We also examined whether time from blood draw to diagnosis of liver cancer (within 4 years of follow-up, *n* = 30 cases; within 8-years of follow-up, *n* = 63 cases; or within 12-years of follow-up, *n* = 112 cases) affected associations between antibodies and liver cancer risk. Finally, we conducted an analysis among participants with no missing data and the results remained the same (data not shown).

## Results

Table 1 presents the baseline characteristics of cases and selected controls. The individuals who developed liver cancer were more likely to be ever smokers and to have a greater BMI than the controls at recruitment (Table 1).

**Table 1:** Characteristics of liver cancer cases and controls selected from PLCO.

	Controls (*N* = 382)	Cases (*N* = 191)
Sex		
Male	278 (72.8%)	139 (72.8%)
Female	104 (27.2%)	52 (27.2%)
Age		
<60 years	112 (29.3%)	56 (29.3%)
60-<65 years	120 (31.4%)	60 (31.4%)
65-<70 years	94 (24.6%)	47 (24.6%)
≥70 years	56 (14.7%)	28 (14.7%)
Race		
White	320 (83.8%)	160 (83.8%)
Black	18 (4.7%)	9 (4.7%)
Hispanic	22 (5.8%)	11 (5.8%)
Asian	20 (5.2%)	10 (5.2%)
Other	2 (0.5%)	1 (0.5%)
Smoking status		
Never	161 (42.1%)	63 (33.0%)
Former	182 (47.6%)	92 (48.2%)
Current	38 (9.9%)	36 (18.8%)
Unknown	1 (0.3%)	0 (0.0%)
Education		
Less Than 8 Years	0 (0.0%)	2 (1.0%)
8–11 Years	27 (7.1%)	19 (9.9%)
12 Years or Completed High School	85 (22.3%)	39 (20.4%)
Post High School Training Other than College	46 (12.0%)	26 (13.6%)
Some College	76 (19.9%)	42 (22.0%)
College Graduate	61 (16.0%)	29 (15.2%)
Postgraduate	85 (22.3%)	33 (17.3%)
Unknown	2 (0.5%)	1 (0.5%)
Body mass index		
<25 kg/m^2^	118 (30.9%)	44 (23.0%)
25-<30 kg/m^2^	174 (45.5%)	79 (41.4%)
≥30 kg/m^2^	83 (21.7%)	63 (33.0%)
Unknown	7 (1.8%)	5 (2.6%)
Alcohol intake, g/day – mean (SD)	12.3 (21.8)	18.4 (41.5)
Coffee intake, g/day – mean (SD)	980 (1173)	847 (850)
Hepatitis C Virus Serology Positive	5 (1.3%)	22 (11.5%)
Hepatitis B Virus Surface Antigen Positive	2 (0.5%)	4 (2.1%)

Values are N (%) unless otherwise indicated.

PLCO, prostate, lung, colorectal, and ovarian screening trial study, SD, standard deviation.

**Fig. 1:**
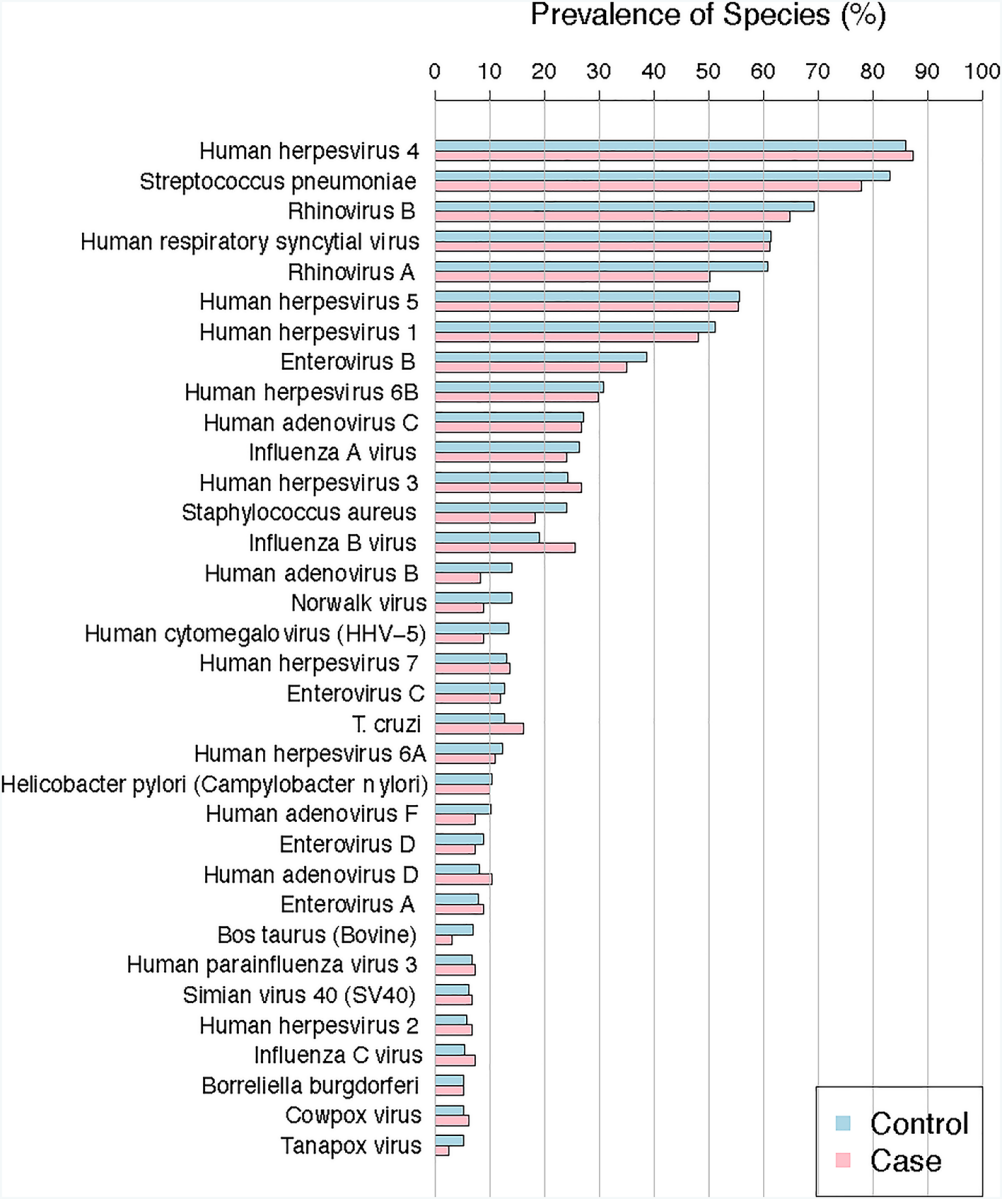
Prevalence of detected antibodies (e.g., viral or bacterial species) separated by liver cancer case and control status. This figure presents the most prevalent antibodies detected by liver cancer case (red) and control (blue) status.

The most common antibodies (at the species level) separated by case/control status are presented in Fig. 1. Human herpes virus 4, *Streptococcus pneumoniae*, and rhinovirus B were the most common pathogenic infections among all participants. Supplementary Fig. 1 presents the prevalence of detected viral organisms, bacterial, or allergen antibodies by case/control status. The greatest differences between covariates and detected antibodies are presented in Supplementary Table 1. Women were more likely to have antibodies against human herpesvirus 6B and human herpesvirus 5 than were men, and White individuals were more likely to have antibodies against rhinovirus B than were non-White individuals (Supplementary Table 1).

The number of detected antibodies in liver cancer cases and controls were not significantly different (mean of 12.0 in controls versus 12.5 in cases, *p* = 0.58). Moreover, when comparing cases and controls, no significant differences were observed between prevalence of specific detected antibodies after correction for multiple testing (Supplementary Fig. 2). However, the most significant viral species detected between cases and controls was HCV (*p* = 0.0032, FDR corrected = 0.35), which was present in 23 participants, of whom 15 were liver cancer cases. When testing for differences between cases and controls, HCV was found to be associated with a OR of 3.98 (95% CI: 1.59–9.99) for liver cancer, although was not statistically significant after correction for multiple testing. Table 2 presents the OR and 95% CI for the top 10 antibodies associated with liver cancer risk.

**Table 2:** Odds ratios and 95% confidence intervals for the top 10 antibodies associated with liver cancer risk.

Antibody	Odds Ratio (95% CI)	*P*-value	FDR corrected *p*-value
Hepatitis C virus	3.98 (1.59–9.99)	0.0032	0.35
Rhinovirus A	0.59 (0.39–0.88)	0.010	0.52
Human Adenovirus B	0.43 (0.21–0.86)	0.017	0.61
Norwalk virus	0.49 (0.26–0.91)	0.023	0.63
Hepatitis B virus	2.55 (0.98–6.59)	0.054	0.99
Influenza B virus	1.57 (0.98–2.51)	0.059	0.99
Bos Taurus Bovine	0.41 (0.15–1.10)	0.077	0.99
Staphylococcus aureus	0.65 (0.40–1.06)	0.084	0.99
Cytomegalovirus (HHV-5)	0.60 (0.33–1.11)	0.11	0.99
Vaccinia virus	1.87 (0.86–4.05)	0.11	0.99

Odds ratios and 95% confidence intervals (CI) obtained from multivariable conditional logistic regression conditioned on age, sex, ethnicity, and time of blood draw, and further adjusted for body mass index, alcohol intake, diabetes status, education, smoking, and coffee intake.

Odds ratios and 95% CI determined for each antibody from a total sample of 573 participants.

CI, confidence intervals; FDR, false-discovery rate.

The concordance of anti-HCV detected by serology or by VirScan is presented in Supplementary Table 2. In total, 12 participants were concordantly assigned between the two methods as being HCV(+). Using serology as the gold standard test, the sensitivity for HCV status between the two methods was 44.4% (95% CI: 25.5-64.7%) whereas the specificity was 98.0% (95% CI:96.4-99.0%). When comparing the number of viral species detected between HCV (+) and HCV (-) participants, persons who were HCV (+) had an average of 4.1 more species than those who were HCV (-) although this comparison was not statistically significant (*p* = 0.072; Supplementary Fig. 3). Very few participants (< 2%) were detected as being HBV positive using VirScan.

Supplementary Fig. 4 presents a heatmap of the correlation coefficients between 109 species (17 were non-viral antibodies, of which 8 were bacterial) based on their presence/absence status. No strong correlations were found. There were only a few weak positive correlations within the two small clusters (top right and bottom left); Human herpes virus 1, 4, and 5 and *Streptococcus pneumoniae* were the species that were most strongly correlated with one another.

**Fig. 2:**
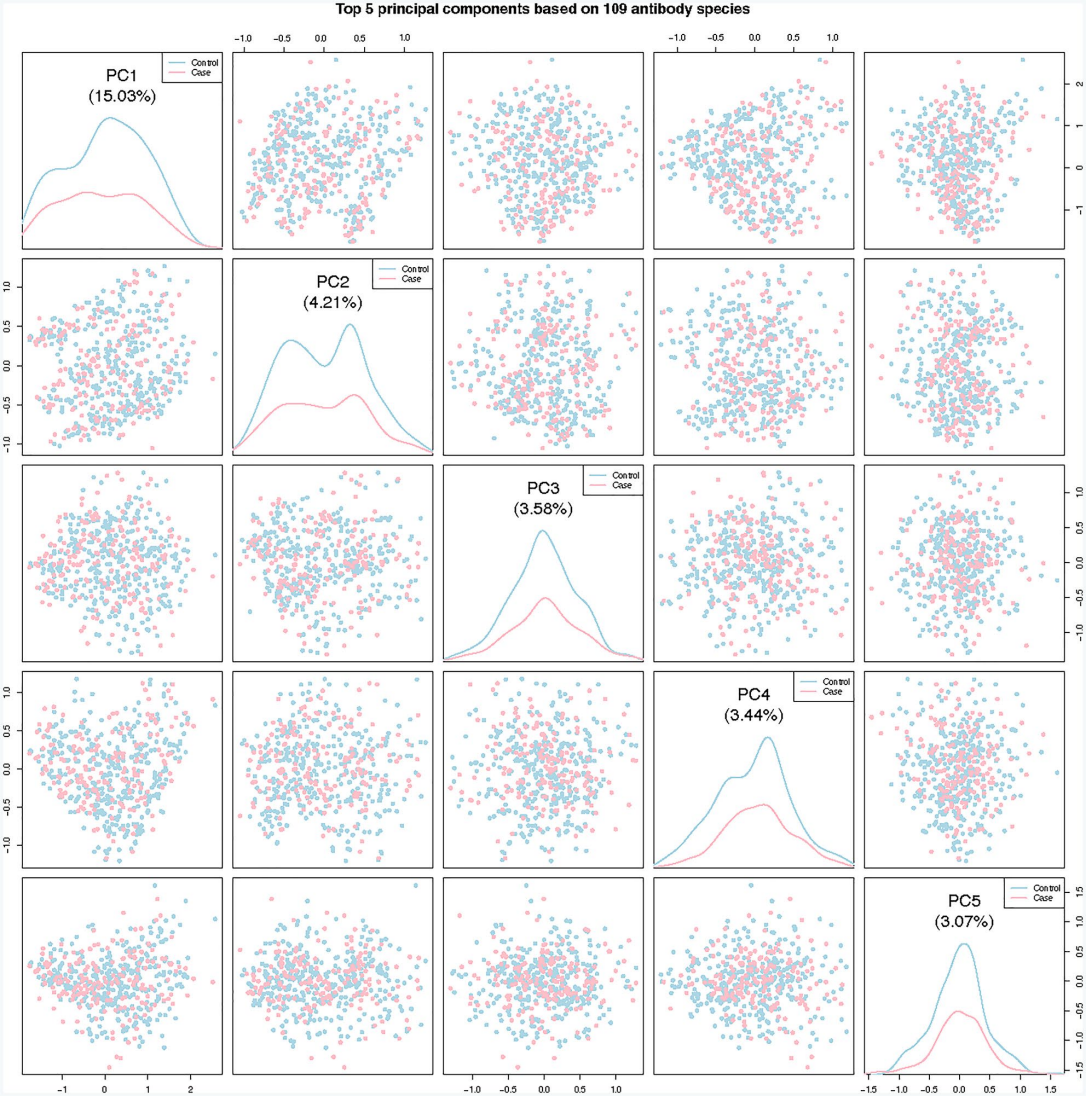
Scatter plot of the top five principal components based on the presence and absence of 109 antibodies between liver cancer cases and controls. Each dot represents a participant (red: cases; blue: controls) and the density lines in the diagonal panels show the distributions of the corresponding principal component in cases/controls.

Supplementary Fig. 5 presents an unsupervised hierarchical clustering of the 109 detected antibodies among all the participants. The clustering results yielded two groups of participants and three groups of virus species. Generally, the main difference between the two groups of participants is the total number of the antibodies/species detected (top: mean 13.3 species (SD:5.4) vs. bottom: mean 3.9 species (SD:2.7)). Specifically, most of the top significantly different detected antibodies are located at the two small clusters (left and right) in the heatmap (Supplementary Fig. 5). However, no significant differences were observed by case/control status between the two groups of the participants. Ordination results – using the top five principal components from the principal component analysis of 109 detected antibodies– are presented in Fig. 2. No significant differences between cases and controls were observed.

**Fig. 3:**
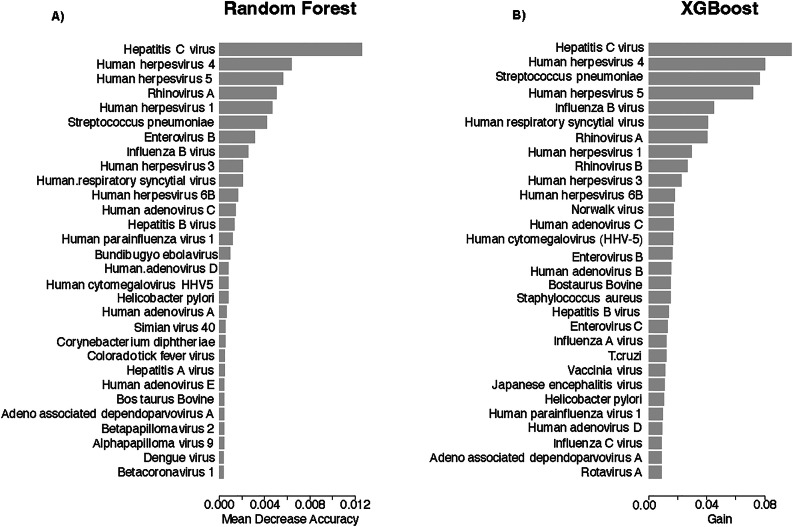
Top important features predicting liver cancer case or control status using (**A**) random forest, or (**B**) XGBoost.

Three machine learning models (LASSO / random forest / XGBoost) were applied to evaluate the performance of the prediction of the case/control status based on the observed Z-sores. The AUC for LASSO was 0.53 (95% CI: 0.41–0.66), for random forest was 0.52 (95% CI: 0.38–0.64), and for XGBoost, was 0.54 (95% CI: 0.40–0.66). Figure 3 shows the most important features selected by random forest and XGBoost. In both random forest and XGBoost, HCV was selected as the top feature.

## Sensitivity analyses

In sensitivity analyses restricted to HCC (119 cases and 238 controls), no antibodies against infectious agents (measured via VirScan), except HCV, were significantly different between cases and controls. Supplementary Table 3 presents the OR and 95% CI for the top 10 antibodies associated with HCC risk. The association between HCV and risk became more significant with an odds ratio of 23.16 (95% CI:4.56–117.7), which was statistically significant after correction for multiple testing (p-value = 0.00015; FDR = 0.0016). Prediction models restricted to HCC improved slightly, with the AUC for random forest being 0.55 (95% CI: 0.43–0.68) and XGBoost 0.55 (95% CI: 0.44–0.67).

In analyses that restricted cases diagnosed within 4-years, 8-years, or 12-years of follow-up, associations remained largely the same and detected antibodies did not differently predict liver cancer cases from our main analyses (AUC ~ 0.55).

## Discussion

In this nested case-control analysis, a history of exposure to infectious agents other than HCV, was not found to be prospectively associated with risk of liver cancer. When applying an antibody exposure signature (primarily from detected viruses) to predict liver cancer development, prediction models were found to perform poorly in differentiating liver cancer cases from controls.

Two previous studies have explored a history of exposure to infectious agents, measured via VirScan, and liver cancer. A United States-based case-control study reported that a viral exposure signature was more common among HCC cases than among controls^10^. Eleven of the 61 epitope binding signals were from HCV strains and 43 viruses were inversely associated with HCC^10^. Similarly, in a case-control study conducted in Thailand, 13 of the 46 viral signals were from HCV and HBV and 21 viral features were inversely associated with HCC^11^. However, as both study results were from a case-control studies, it is not possible to determine whether the viral exposures were present before the development of cancer. Evidence from mouse models has suggested that individuals with cancer may have altered immune function and have weaker immune responses to infections that are tumour-driven^19^. This may be particularly important as differences in viral infections at diagnosis may be a result of poor immune response caused by the tumour immune dysfunction rather than a viral infection that is a causative agent of liver cancer development. However, general circulating IgG antibodies have been found to be similar between individuals with and without cancer^20^ and further research exploring immune changes among individuals with cancer are needed.

Approximately 24-26% of all participants in the aforementioned case-control studies had an HCV or HBV infection. Chronic HCV infection is known to dysregulate the immune system, which may lead to viral chronicity^21^. Although both studies conducted a prospective validation study in 173 individuals using blood samples measured ~ 8.8 years before cancer incidence, the participants were members of a cohort at high-risk of HCC, with 92% being HCV(+) or HBV(+), all of whom were diagnosed with chronic liver disease^10,11^. This is in contrast to the current study where the participants were drawn from a population-based sample and only 4% were HCV(+). The differences in the populations may be particularly important as the individuals may differ on numerous characteristics, which may introduce selection bias. For example, in the current analyses, we observed that HCV(+) individuals were exposed to more viral species than HCV(-) individuals, however, this was not statistically significant potentially due to the small number of HCV(+) participants in the study. Considering this, the same infectious agent signature may not have the same utility or accuracy in a population where HCV and HBV are uncommon.

Chronic HCV is an established causal risk factor for liver cancer, particularly HCC^4,8,22^ and in this analysis, HCV was found to be the only viral signature associated with liver cancer risk. HCV is known to promote liver cancer carcinogenesis by causing chronic inflammation, liver fibrosis, and cirrhosis^23^, which can then lead to HCC. Some evidence has suggested that different HCV genotypes can alter the risk of HCC, in particular, a meta-analysis including 21 studies suggested that HCV-1b had 78% greater risk of HCC than other HCV genotypes^24^. HCV1a and HCV1b are the most common strains in the United States and HCV1b strain was also found to be enriched in HCC cases in a prior case-control analysis utilizing VirScan^10^, however, in the current analysis HCV1b was rare among the participants (~ 2%) likely due to the relative rarity of HCV overall.

Previous evidence has suggested that some infectious agents are associated with a greater risk of obesity^25,26^, and these viral infections may be particularly important for liver cancer risk as adiposity and MASLD are established risk factors for liver cancer^27,28^. In one study that utilised VirScan, human herpesvirus 1 (HSV-1) was found in a sample of Qatari adults to be associated with a greater odds of being obese^26^. In the current study, however, HSV-1 was more common in controls than cases, although the difference was not statistically significant.

### Strengths and limitations

A major strength of the current study was that it was nested within a large, well-characterized prospective study. The liver cancer outcomes were ascertained via linkage to cancer registries and the study participants were tested for HBV and HCV markers by serology. There are also several limitations to consider for the current study. The statistical power to detect small differences in infectious agent exposure between the 191 cases and their 382 controls may have been somewhat limited. In addition, VirScan was shown to have low sensitivity for HCV in comparison to results available for serology. Previous evidence that examined the sensitivity and specificity of VirScan in contrast to a medical report of HCV infection has found varying results^10,12^. Sensitivity was found to be relatively high in the previous case-control study comparing VirScan results to medical records (84%), however, specificity was relatively low (~ 45%), indicating VirScan cut-offs for determining HCV may have a low threshold. In the current analysis there was a relatively small number of participants who were HCV(+), as such, this may have affected the sensitivity of the VirScan results. In a prior examination of 26 HCV(+) Americans, however, sensitivity was reported to be 92%^12^. Further research determining appropriate Z-score cut-offs for HCV infection may be required. Finally, participants in the study provided blood samples ~ 11 years (median) before liver cancer diagnosis of the cases and they may have been exposed to other viruses within that time which could, in theory, be related to their risk of liver cancer. Antibodies can wane over time depending on exposure, therefore the presence of an antibody may reflect a recent exposure or a robust humoral response and how this relates to liver cancer, if at all, is not clear. There is no evidence, however, that levels would preferentially decline among the cases, but not among the controls. Future prospective studies should consider using multiple serum measurements over time to determine if pathogenic infections are associated with liver cancer risk.

## Conclusion

In conclusion, the current study found no evidence that a history of exposure to infectious agents, other than HCV, was associated with development of liver cancer risk. Future research in large prospective studies using serial serum samples over time is needed to confirm these results.

## Electronic supplementary material

Below is the link to the electronic supplementary material.

Supplementary Material 1

## Data Availability

Data from PLCO were obtained from the National Cancer Institute and are available to researchers pending approval from the PLCO Screening Trial Steering Committee. Further details can be found at https://cdas.cancer.gov/plco/. For this present study, data can be made available upon request by contacting the corresponding author.
