# Supplementary material for: Pan-serological antibodies and liver cancer risk: a nested case-control analysis
Source: Sci Rep. 2025 Feb 14;15:5450. doi: 10.1038/s41598-025-89629-2 (PMC11828989; doi:10.1038/s41598-025-89629-2)
Supplement: Supplementary file 1 — Supplementary Material 1 [file 41598_2025_89629_MOESM1_ESM.docx]

**Supplementary Figures**

**Supplementary Figure 1**. Prevalence of presence of viral (organism level), bacterial, or allergen antibodies separated by case and control status.

**Supplementary Figure 2**. QQ Plots of expected and observed p-values for different prevalence of viral species and organisms between cases and controls.

The species of Hepatitis C virus (HCV) and the two organisms of HCV are labeled.

Hepatitis C virus status of participant

**Supplementary Figure 3.** Number of antibodies observed between HCV (-) and HCV (+) participants

P-value from two-sample t-test.


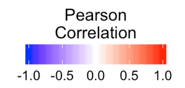


**Supplementary Figure 4**. Heat map of the Spearman’s correlation coefficients between 109 viral, bacterial, or allergen antibodies based on their presence/absence status across all samples.

Presence or absence of species

Species

Participant

**Supplementary Figure 5**. Unsupervised hierarchical clustering of 573 participants by case and control status and 109 detected antibodies

The clustering applied Ward's minimum variance method and Manhattan distance was used to measure the similarity between each pair of participants or species based on the corresponding presence/absence status.

Blue represents that the antibody was not detected in the participants whereas yellow represents that the antibody was detected.

**Supplementary Table 1**. Associations between viral species or organisms and covariates.

| Name of virus or bacterium | Level | Covariate | P-value | FDR | Note* |
| --- | --- | --- | --- | --- | --- |
| Human herpesvirus 6B | species | Sex | 0.00004 | 0.00474 | male 26% vs. female 44% |
| Human herpesvirus 5 | species | Sex | 0.00044 | 0.0528 | male 51% vs. female 68% |
| Human herpesvirus 6B (strain Z29) (HHV-6 variant B) (Human B lymphotropic virus) | organism | Sex | 0.00002 | 0.00491 | male 25% vs. female 44% |
| Human cytomegalovirus (strain AD169) (HHV-5) (Human herpesvirus 5) | organism | Sex | 0.00003 | 0.00634 | male 49% vs. female 68% |
| JC polyomavirus | species | Smoke | 0.00077 | 0.0926 | current 5.4% vs. former/never 0.4% |
| Rhinovirus B | species | Race | 0.00002 | 0.00254 | white 72% vs. other 46% |
| Human herpesvirus 4 | species | Race | 0.00006 | 0.00696 | black 52% vs. other 88% |
| Enterovirus B | species | Race | 0.00038 | 0.0444 | white 41% vs. other 19% |
| Human respiratory syncytial virus | species | Race | 0.00048 | 0.0561 | white 65% vs. other 43% |
| Epstein-Barr virus (strain GDI) (HHV-4) (Human herpesvirus 4) | organism | Race | 0.00002 | 0.00439 | black/Asian 20% vs. other 50% |
| Human respiratory syncytial virus | organism | Race | 0.00032 | 0.0661 | white 62% vs. other 40% |
| Vaccinia virus (strain Copenhagen) (VACV) | organism | BMI | 0.00011 | 0.0226 | presence 23.97 (2.97) vs. absence 27.87 (4.98) |
| Human adenovirus F serotype 40 (HAdV-40) (Human adenovirus 40) | organism | BMI | 0.00014 | 0.0286 | presence 24.68 (3.38) vs. absence 27.94 (5.00) |

* Prevalence of the species/organism in the corresponding categories of sex/smoke/race; mean (SD) of BMI in the presence/absence group of the two organisms.

P values are based on Fisher’s exact test for categorized covariates (Sex/Smoke/Race) and Wilcoxon rank sum test for BMI.

Abbreviations: BMI, body mass index.

**Supplementary Table 2.** Concordance table between antibody HCV serology and VirScan antibody HCV.

|  | **Anti-hepatitis C virus - serology** | |  |
| --- | --- | --- | --- |
| **Hepatitis C Virus - VirScan** | Nonreactive | Reactive | Total |
| Absence | 533 | 15 | 548 |
| Presence | 11 | 12 | 23 |
| Total | 544 | 27 | 571 |
| Two participants did not have HCV serology results and therefore were not included.  Abbreviations: HCV, hepatitis C virus. | | | |

**Supplementary Table 3**. Odds ratios and 95% confidence intervals for the top 10 antibodies associated with hepatocellular cancer risk

| **Antibody** | **Odds Ratio (95% CI)** | **P-value** | **FDR corrected p-value** |
| --- | --- | --- | --- |
| **Hepatitis C virus** | **23.16 (4.56-117.68)** | **0.00015** | **0.016** |
| Rhinovirus A | 0.59 (0.35-1.01) | 0.055 | 0.99 |
| Human Adenovirus B | 0.39 (0.15-1.04) | 0.059 | 0.99 |
| Rubella virus | 5.23 (0.88-31.18) | 0.069 | 0.99 |
| Cosavirus A | 3.36 (0.89-12.74) | 0.074 | 0.99 |
| Human Adenovirus F | 0.34 (0.10-1.15) | 0.082 | 0.99 |
| Norwalk virus | 0.51 (0.23-1.14) | 0.101 | 0.99 |
| Middle East respiratory syndrome coronavirus  (MERS-CoV) | 3.60 (0.77-16.97) | 0.105 | 0.99 |
| Bostaurus Bovine | 0.36 (0.08-1.57) | 0.17 | 0.99 |
| Rhinovirus B | 0.70 (0.42-1.17) | 0.17 | 0.99 |

Odds ratios and 95% confidence intervals (CI) obtained from multivariable conditional logistic regression matched on age, sex, ethnicity, and time of blood draw, and further adjusted for body mass index, alcohol intake, diabetes status, education, smoking, and coffee intake.

Models are restricted to hepatocellular carcinoma cases and matched controls (119 cases and 238 controls).

Abbreviations: CI, confidence intervals; FDR, false-discovery rate
